# Diabetes Mellitus on YouTube: A Cross-Sectional Observational Study to Assess the Quality and Reliability of Videos

**DOI:** 10.7759/cureus.43704

**Published:** 2023-08-18

**Authors:** Maneeth Mylavarapu, Darshilkumar Maheta, Shereece Clarke, Kashish Parmar, Majaazuddin Mohammed, Chaitanya Sai Vuyyuru

**Affiliations:** 1 Department of Public Health, Adelphi University, Garden City, USA; 2 Department of Public Health, New York Medical College, New York, USA; 3 Department of Obstetrics and Gynaecology, University of the West Indies, Montego Bay, JAM; 4 Department of Internal Medicine, GMERS Medical College, Valsad, Valsad, IND; 5 Department of Internal Medicine, Shadan Institute of Medical Sciences, Hyderabad, IND; 6 Department of Internal Medicine, St. Martinus University Faculty of Medicine, Willemstad, CUW

**Keywords:** digital health, healthcare information, reliability score, global quality score, youtube videos, diabetes mellitus

## Abstract

Introduction

Diabetes mellitus (DM) encompasses a group of heterogeneous, chronic, and non-communicable diseases characterized by an increase in blood glucose levels. As it has become easily accessible for patients to know about their symptoms and treatment of diseases, it is of utmost importance that reliable information is conveyed on the internet. If not managed appropriately, it may result in the dissemination of false information, leading to risky practices and incorrect treatment, further resulting in detrimental consequences.

Aim

To assess the quality and reliability of information related to DM on YouTube.

Methodology

A cross-sectional observational study was conducted in April 2023, wherein top YouTube videos related to 'diabetes' were analyzed for baseline characteristics, type of uploader, as well as quality and reliability using Global Quality Score (GQS) and Reliability Score (DISCEN), respectively. Statistical analysis was performed using SPSS software.

Results

A total of 87 videos were evaluated in the study. Unfortunately, only 21% of those were uploaded by doctors. The median Video Power Index (VPI) for videos uploaded by other sources was the highest (184.7), and the lowest was for videos uploaded by hospitals (12.6), and this was statistically significant (p = 0.038). The median GQS was highest for videos uploaded by doctors (4) and lowest for videos uploaded by others (3.5). The reliability score was higher in videos uploaded by healthcare organizations (4), which was not significant (p > 0.05).

Conclusions

Videos uploaded by physicians and healthcare organizations contained reliable information with a high global quality score. Videos uploaded by sources other than doctors and healthcare professionals should consult physicians, as self-diagnosis or self-treatment can lead to potential harm to patients.

## Introduction

Diabetes mellitus (DM) encompasses a group of heterogeneous, chronic, and non-communicable diseases characterized by an increase in blood glucose levels [1]. This complex metabolic illness is linked to an increased risk of both microvascular and macrovascular disease. According to the American Diabetic Association (ADA), diabetes can be broadly classified into type 1, type 2, gestational diabetes mellitus, and specific types of diabetes due to other causes. Type 1 DM occurs when the immune system attacks and kills the cells in the pancreas that make insulin, resulting from a genetic predisposition that often presents earlier in life. Type 2 DM, on the other hand, occurs when there is insulin resistance and is linked to one's daily lifestyle decisions [2]. The prevalence of type 2 DM is increasing at an alarming rate worldwide. However, efforts are being made to educate patients on lifestyle changes and treatment options for this disease.

YouTube has become an easily accessible and fast-paced resource for many patients seeking to learn about their symptoms and treatment of diabetes [3]. However, the quality of content in these videos can be questionable if not uploaded by a reliable source, leading to the circulation of misleading information. This is clinically significant as the information conveyed using this platform is very influential. Many patients who have the disease themselves often share their experiences, and viewers are now able to relate to the content shared. Healthcare professionals, such as doctors and healthcare organizations, also seek to spread awareness through this forum to educate patients on when to seek medical attention [4]. It is of utmost importance that reliable information is conveyed in these videos, as the grave complications of diabetes, if not managed appropriately, may result in increased severity of the disease [5]. The aim of this study is to assess the quality and reliability of information present on YouTube regarding DM using the Global Quality Score (GQS) and Reliability Score (DISCERN).

## Materials and methods

This web-based, cross-sectional observational study conducted in April 2023 does not involve human participation. The study was conducted on a single day, April 19. First, a questionnaire was created on Google Forms that included predetermined criteria related to videos such as the number of likes, dislikes, views, source of video uploaded, and comments. Then, we assessed the content of the videos that were found using keywords like diabetes, diabetes treatment, diabetes prevention, diabetes cause, diabetes cure, and diabetes diet.

All the authors evaluated 15 videos, and any repeated entries were deleted. Then, screening for inclusion and exclusion criteria was done. In order to be included, videos should have specifically contained information about diabetes and should have been presented in the English language. All the videos that did not contain information about diabetes or were presented in any language other than English were excluded. Also, videos that were shorter than one minute or longer than 15 minutes in duration were excluded. The next step involved assessing the quality and reliability of the videos using GQS and Reliability Score [6]. The responses were recorded and transferred to Google Sheets. Finally, statistical analysis was done using SPSS software (IBM Corp., Armonk, NY).

## Results

A total of 87 videos were evaluated in this study. After meticulous screening for inclusion and exclusion criteria and the deletion of duplicates, 62 videos were included in the study. The total number of views was 28,243,110, the total number of likes was 426,667, the dislikes were 15,480, and the comments were 29,695. Over 87% of the videos were uploaded more than a year ago. The majority of the videos (35.5%) were posted by sources other than doctors, hospitals, healthcare organizations, and news agencies. Unfortunately, only 21% of the videos were posted by doctors. The detailed characteristics of the analyzed videos are depicted in Table 1. According to our study, 62.90% of the videos discussed causes or aetiologies, 40.32% discussed symptoms, 45.16% discussed vaccinations or prevention, 50.00% discussed complications, 19.35% discussed investigations or tests, 19.35% discussed mortality, 33.87% discussed rehabilitation, and 14.52% discussed support groups (Table 2).

**Table 1:** Characteristics of the YouTube videos analyzed (N=13)

Time since the video uploaded	N (%)
More than a week to one month (7 - 30 days old)	01 (01.6%)
More than a month to six months (31 - 180 days old)	06 (09.7%)
More than six months to last one year (180 - 365 days)	01 (01.6%)
More than one year (> 365 days)	54 (87.1%)
Popularity	N
Total no. of views	28243110
Total no. of likes	426667
Total no. of dislikes	15480
Total no. of comments	29695
Type of uploader	N (%)
Doctor	13 (21.0%)
Hospital	10 (16.1%)
Healthcare organization	12 (19.4%)
News channel	05 (08.1%)
Other	22 (35.5%)

**Table 2:** Information about being "diabetic" shared by the YouTube videos (N=228)

Information	N (%)
Description of SYMPTOMS	25 (40.32%)
Cause/etiology?	39 (62.90%)
Investigations/tests	12 (19.35%)
Prevention/vaccines	28 (45.16%)
Treatment	39 (62.90%)
Mortality	12 (19.35%)
Rehabilitation	21 (33.87%)
Complications	31 (50.00%)
Support groups	09 (14.52%)
People/patients sharing their own experience	07 (11.29%)
Parent Sharing Their Experience With Their Family Members	03 (04.84%)
The Post Has Promotional Content By Pharmaceutical Companies or by Doctors	02 (03.23%)

The Global Quality Score (GQS), Reliability Score (RS), and Video Power Index (VPI) are compared based on the uploading of the video in (Table 3). The median VPI for videos uploaded by other sources was highest (184.7) and lowest for videos uploaded by hospitals (12.6), and this difference was statistically significant (p= 0.038). The median GQS was highest for videos uploaded by doctors (4) and lowest for videos uploaded by other sources (3.5). However, for the Reliability Score, the median score was highest for videos uploaded by healthcare organizations (4) and lowest for videos uploaded by news agencies (3). However, these differences were not statistically significant.

**Table 3:** Comparison of VPI, GQS, and RS based on the type of uploader (N=62) n, sample size; VPI, Video Power Index; GQS, Global Quality Score; RS, Reliability Score All cells contain values expressed as: median (IQ1, IQ3) Statistical analysis used: Kruskal-Wallis test

	Doctors (n=13)	Hospitals (n=10)	Healthcare organization (n=12)	News Agency (n=5)	Others (n=22)	P value
VPI	84.01 (33.96, 220.90)	12.595 (8.17, 34.00)	26.49 (6.87, 181.67)	49.4 (3.36, 1997.97)	184.71 (28.16, 849.18)	0. 038
GQS	4 (2.5, 5)	4 (2, 4.25)	4 (3.25, 5)	3 (2.5, 4.5)	3.5 (2.75, 4)	0. 412
RS	3 (2, 4)	3.5 (2, 4.25)	4 (3.25, 4)	3 (2.5, 4)	3.5 (2, 4)	0. 302

## Discussion

Diabetes mellitus (DM) is a complex condition that requires patients to make several daily choices encompassing their diet, exercise routine, and medication. In addition, it demands that individuals possess a range of self-management abilities [7]. The ease with which material can be created using web tools has transformed how people communicate. Patients increasingly use the internet to understand their medical illnesses and treatments better and make informed healthcare decisions. Studies have documented the proven potential of video-based platforms in improving insights into multiple facets of the disease, including the etiology, clinical presentations, signs and symptoms, diagnostic procedures, treatment and management procedures, potential complications, and preventive strategies [8]. Among these web-based platforms, YouTube has emerged as a prominent platform for the dissemination of health information to both professionals and patients alike, owing to its distinctive advantages as an educational medium.

In the context of this study, we utilized the GQS, RS, and VPI scores as tools for assessing the quality, reliability, and popularity of the content from each of the uploaders. Osman et al. reported that the quality of health-related content on YouTube was below average [9]. Conversely, in our study, the average GQS score was 3.6, implying the higher quality of the videos on YouTube compared to previous research.

Pertaining to the quality of videos based on the type of uploader, we found that videos uploaded by doctors and hospitals had the highest quality. Consistent with the findings of our study, Diers et al., in their evaluation of the usefulness of YouTube videos on asthma, found that videos posted by doctors had the highest quality [10]. Similar conclusions were also drawn by Holge et al. in their study regarding the quality and reliability of YouTube videos on myocardial infarction [11]. Complementary trends were also found on other social media and web-based platforms. A study by Aiman et al., which focused on evaluating the quality and reliability of the content on obesity on Instagram, reported that the posts by doctors and healthcare organizations on obesity had the highest quality and reliability scores [12].

According to our analysis, VPI was higher in videos posted by sources other than doctors, healthcare organizations, hospitals, and news agencies (184.71) compared to those posted by doctors (84.01), healthcare organizations (26.49), hospitals (12.595), and news agencies (49.4). A similar study on rotator cuff repair by Celik et al. found that VPI was higher in videos posted by doctors [13]. Furthermore, in our analysis, most of the videos (62.90%) comprised information on treatment for diabetes. In contrast, a comparative study by Kaya et al. on hypertension revealed that only 39.4% of YouTube videos contained information about pharmacological treatment [14].

Regarding RS, we found that the reliability index was higher in videos by hospitals and healthcare organizations. In contrast to our study findings, existing literature reports that content posted by doctors has the highest quality and reliability across social media and web-based platforms [15,16].

Limitations

One of the main limitations of our study was that we only analyzed videos in English. Second, the YouTube platform is very dynamic, and the results may change by adding new videos or removing old ones. Finally, the limited sample size could be another limitation; however, it is unusual for any viewer to go through 50+ videos on YouTube.

## Conclusions

Responsible individuals or organizations should upload verified information that is easily understandable by the general population and provides all relevant and necessary information about diabetes. The videos should contain reliable information with a high global quality score. Additionally, it is important to mention in these videos that self-diagnosis and self-treatment can potentially harm the patient, and one should always consult a doctor or physician for proper diagnosis and treatment.
